# Assessing coral sperm motility

**DOI:** 10.1038/s41598-020-79732-x

**Published:** 2021-01-08

**Authors:** Nikolas Zuchowicz, Jonathan Daly, Jessica Bouwmeester, Claire Lager, E. Michael Henley, C. Isabel Nuñez Lendo, Mary Hagedorn

**Affiliations:** 1 Center for Species Survival, Smithsonian Conservation Biology Institute, Smithsonian Institution, Front Royal, VA USA; 2 Hawaii Institute of Marine Biology, University of Hawaii, Kaneohe, HI USA; 3 Taronga Conservation Society, Sydney, NSW Australia; 4 Aquatic Germplasm and Genetic Resources Center, Louisiana State University Agricultural Center, 2288 Gourrier Avenue, Baton Rouge, LA 70820 USA

**Keywords:** Animal physiology, Marine biology, Reproductive biology

## Abstract

The declining reproductive viability of corals threatens their ability to adapt to changing ocean conditions. It is vital that we monitor this viability quantitatively and comparatively. Computer-assisted sperm analysis (CASA) systems offer in-depth analysis used regularly for domestic and wildlife species, but not yet for coral. This study proposes quality control procedures and CASA settings that are effective for coral sperm analysis. To resolve disparities between CASA measurements and evaluations by eye, two negative effects on motility had to be resolved, slide adhesion (procedural) and sperm dilution (biological). We showed that the addition of bovine serum albumin, or caffeine, or both to fresh sperm reduced adhesion in the CASA cassettes, improved motility and motile sperm concentration (*P* < 0.0001), yet these additions did not affect measurements of total sperm concentration. Diluting coral sperm reduced sperm motility (*P* = 0.039), especially from heat-stressed corals. We found CASA concentration counts comparable to haemocytometer and flow cytometer measures (*P* = 0.54). We also found that motile sperm per egg is a useful predictor of fertilisation success, using cryopreserved sperm. Standard measurements of coral reproductive characteristics inform our understanding of the impacts of climate change on reef populations; this study provides a benchmark to begin this comparative work.

## Introduction

Around the world, coral reefs are in danger^1^. They support a vast ecosystem^2^ that provides billions of dollars annually in material benefits to humans through coastal protection, fisheries, and tourism^3^. Climate change, land-based pollution, and commercial overexploitation threaten both the reefs and the human and natural communities that depend on them^4^. Coral bleaching, disease, and death are likely to increase in prevalence as the ocean warms and acidifies^1,5–7^.

Sexual reproduction in coral generates new, better-adapted genotypes capable of withstanding the changing conditions in the ocean. Many studies have determined that bleaching impacts reproduction in coral in many ways, and these effects often last more than one spawning season^8–10^. On Heron Island during the global bleaching event in March 1998, some coral colonies bleached, while others did not. Six months later, the corals that recovered from bleaching had fewer polyps containing eggs and testes and the existing eggs were smaller than in the unbleached colonies^8^. The following year, no reproduction was reported in the corals that recovered from bleaching^8^. Moreover, an 11-year study in Panama demonstrated the stressful long-term impacts of warming: reproduction was diminished in both bleached and unbleached corals^9^. Recently, our laboratory examined a number of reproductive parameters in a longitudinal study of coral in Hawaii. In coral post-bleaching, nearly a 50% decrease in mean fresh sperm motility in *Montipora capitata* and *Lobactis scutaria* (formerly *Fungia scutaria*) and an overall reduction of egg volume in *L. scutaria* was observed^10^.

Coral reproductive biologists rarely examine the health or the concentration of coral sperm during in vitro reproduction, relying mostly on a qualitative visual assessment of their test sperm solution. Under non-bleaching, minimal-stress conditions, judging concentration by eye has worked well to produce robust fertilisation success across several coral species^11–13^. With decreasing quality of reproduction, this approach no longer assures success; it is therefore critical to develop robust, rapid and repeatable direct assessment methods for sperm quality. Ex situ assisted reproduction methods that cryopreserve and store coral sperm samples^14^ and produce new genotypes with frozen and thawed sperm^11^ depend on consistent post-thaw sperm motility and well-defined sperm-to-egg ratios for consistent in vitro results. Standardised assessment methods will provide comparable results across reefs globally, enabling effective ex situ conservation action and enhancing our understanding of the ongoing changes to coral reproductive health.

Assessments of coral sperm motility are hampered by the limited time window for sexual reproduction each year, remote reef locations that inhibit access to material, and the lack of trained personnel, which leads to inconsistencies in reporting of motility. Computer-assisted sperm analysis (CASA) offers to automate and standardise much of the time-consuming work that limits sample throughput during a successful spawn. Several different commercial and non-commercial systems are currently deployed in the reproductive research community^15^. We make use of the CEROS II (Hamilton Thorne), a commercial platform that can analyse a sperm sample under a phase or darkfield microscope in a few seconds. CASA already plays a central role in agriculture and in assessing many wildlife species^16^, including some invertebrates^17–20^, but has yet to see widespread use for coral.

This study describes the first comprehensive testing and use of CASA to assess coral sperm. We have conducted a five-year assessment of CASA on a variety of coral species and present guidelines for conducting fast, accurate CASA analysis with the hope of expanding the use of CASA to many reef systems globally. This study was necessarily prolonged by the natural history of coral: they have some of the most restricted sexual reproduction in the animal kingdom, most species spawning only three to nine nights each year^21,22^. This allows a couple of hours each evening before sperm quality drops, yielding a total of < 18 h per year to determine optimal protocols for each species that is available to us^23,24^. There is no one right approach in trying to determine coral sperm concentration or motility; all counting methods have limited concentration ranges, and errors appear if the sperm concentration is too high or too low. The key to using CASA effectively for coral sperm is to understand the potential pitfalls and to be consistent in sample handling, allowing accurate comparison from season to season and place to place.

In this paper, we describe the combined use of phase microscopy, haemocytometer measures, flow cytometry, and CASA to measure several aspects of coral sperm motility. We found that there were many aspects of coral sperm motility and the integration of CASA analysis into standard reproductive analyses that must be taken into consideration. These included problems with sperm sticking to the CASA slides, effects of sperm dilution ratios on other measured parameters, concerns that sperm concentration measurements might differ among CASA, haemocytometer, and flow cytometer, the difficulty of parametrising meaningful motility categories, and the effects of adding bovine serum albumin and caffeine to sperm samples. Although we assessed coral sperm over the course of six years, we initially collected data that was not consistent with our experience of measuring sperm by eye with a phase microscope and haemocytometer, and so had to make many adjustments to our protocol. Corals spawn infrequently, so this took a great deal of time. We are therefore only presenting the last two years of our data collection, demonstrating a feasible CASA protocol for capturing key motility parameters of coral sperm. Our hope is that this paper will enable rapid, accurate measurement of coral sperm in laboratories around the world, to include future studies checking correlations between such parameters and fertilisation outcomes.

## Results

### Coral sperm detection with CASA

Overall, it was necessary to keep parameters fairly wide (e.g., cell head size 5–150 µm^2^) to ensure consistent sperm detection. We had trouble getting the CASA system to pick up sperm tails adequately; therefore, we had to turn off tail detection (which normally appears in the CASA software as red pixels) and set *Static Tail Filter* = *False*. Leaving *Static Tail Filter* = *True* leads to underestimation of concentration and overestimation of motility: no tails are visible, so no static cells at all are counted. Due to our use of darkfield rather than phase, the photometer calibration tool built into the software was not used; our darkfield setup had too dim a field for the working range of the photometer.

Coral reproduction takes place in dusty, humid, salty field environments which compromised our CASA cassettes. Dust falling onto CASA cassettes or salt crystalizing on them produced light blooms and haloes in the camera field of view and was impossible to clean effectively from the closed cell. Because of this, the *Cell Travel Max* parameter (i.e. the maximum distance that a cell may travel between video frames and still be considered by the software as the same cell) in some cases was lowered from 10 to 5 µm or even less; this helped eliminate false cell path detections in light blooms. However, the CASA operator must visually check the analysed videos to ensure that this does not split up genuine sperm paths, especially where sperm have high progressive velocity. For example, *Cell Travel Max* = 5 µm will falsely disconnect the paths of sperm travelling close to a curvilinear velocity (VCL) of 5 µm × 60 Hz = 300 µm∙s^−1^. Generally, maintaining the cassettes in a sealed container with a desiccant solved this issue.

The cut-off values for binning sperm by motility (static vs. slow; slow vs. motile; motile vs. progressive) were determined qualitatively by eye and experience with a particular species. Some species, such as *Acropora* spp., have almost mammalian-type sperm motilities with rapid straight-line motion, whereas other species, such as *L. scutaria*, can just wiggle in some months, and progressively move in others. This must be checked for each species and sometimes month-to-month as characteristics change. Therefore, sperm were binned into the static category if they moved less than 80% of their head diameter from their initial position during the 0.75 s video capture (this corresponds to *Static Algorithm* = *Width Multiplier* and *Static Width Multiplier* = 0.8). Sperm were considered slow if they moved at less than 20 µm/s, motile if they moved at between 20 and 80 µm/s, and progressive if they moved at greater than 80 µm/s.

Additional microscope and CASA settings had to be modified for our own microscopes rather than the ones included in the standard Hamilton Thorne platform and can be found in Supplementary Information I. The full list of detailed CASA settings that we used can be found in Supplementary Table S1.

### Considerations for assessing sperm motility

#### Use of BSA for sperm motility assessments with CASA

**Figure 1:**
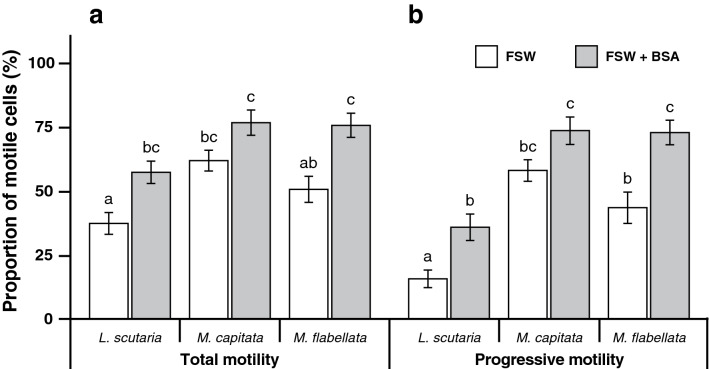
Computer-assisted sperm analysis (CASA) assessment of total (**a**) and progressive (**b**) sperm motilities in samples from *Lobactis scutaria* (n = 20), *Montipora capitata* (n = 9), and *Montipora flabellata* (n = 15) in the presence of filtered seawater (FSW) and filtered seawater with bovine serum albumen (FSW + BSA). Data are presented as mean ± SE, and in (**a** and **b**) in each panel (reflective of a single species), columns with the same letter are not significantly different (*P* < 0.05).

Coral sperm tended to stick to the CASA slides, leading to heavy underestimation of sperm motility. We tested whether the addition of BSA reduced this stickiness and allowed for consistent readings of sperm motility. Initially, sperm from the Hawaiian corals *L. scutaria* (n = 20), *M. capitata* (n = 9), and *M. flabellata* (n = 15) were assessed as usual in 0.22-µm-filtered seawater (FSW), then treated with BSA and re-assessed to determine the overall effects of this addition (Fig. 1). The BSA significantly improved the total mean motility from an average range of 8‒30% to 15‒47% (mixed-model ANOVA, F_(1,43)_ = 57.61, P < 0.0001) and the mean progressive motility by ~ 16‒30% (mixed-model ANOVA, F_(1, 43)_ = 49.49, P < 0.0001) with significant increases noted in *L. scutaria* and in *M. flabellata* (Fig. 1; Supplementary Tables S2, S3). In summary, BSA was extremely beneficial for coral motility assessments with CASA.

#### BSA and caffeine tests

**Figure 2:**
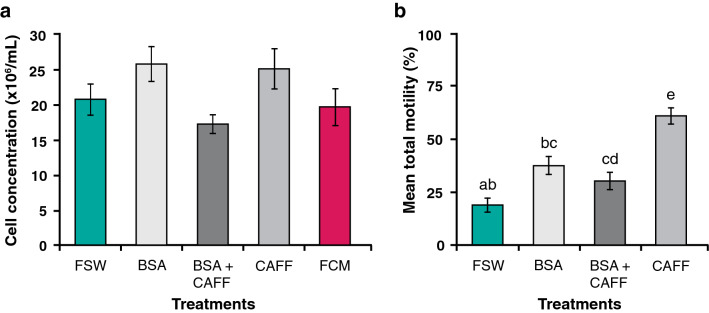
*Lobactis scutaria* sperm samples (n = 14) were treated with four different solutions for 5 min, and the total concentration and corresponding sperm motility were measured on a CASA. A flow cytometer was used to ground-truth the fresh total concentration of the sperm samples. (**a**) The total concentration of the *L. scutaria* sperm was measured by CASA under four different conditions, using FSW (filtered seawater); BSA (Bovine Serum Albumin); BSA-CAFF (Bovine Serum Albumin and Caffeine); and CAFF (Caffeine). No difference was observed amongst the means (ANOVA; *P* = 0.08, F = 2.22), suggesting that the treatments had no effect on the measured mean total concentrations. (**b**) The resulting mean total sperm motilities were different based on treatments; CAFF was the highest with almost double the mean motility (ANOVA; *P* < 0.001, F = 18.1). The small letters indicate treatments that are different. Error bars are SE.

In exploring the effects of BSA on adhesion to CASA slides, we undertook a further trial with *L. scutaria* (n = 14) to evaluate a more comprehensive set of treatments. In doing so, we wanted to ground-truth with flow cytometry measurements to check whether such treatments artificially decreased the observed concentration of cells in a slide. The treatments used were FSW, BSA, BSA + caffeine, and caffeine alone. We determined that there was no difference in their measured cell concentrations (*p* = 0.08, Fig. 2a) across the treatments, but there was a difference amongst the treatments in their mean total motilities (ANOVA; *P* < 0.001, F = 18.1; Fig. 2b). FSW had the lowest mean total motility (19%) and BSA + caffeine had the highest mean total motility (60%) for each individual tested. If there had been a difference in the concentration across treatments, this could have affected the motility measurements, making the treatment unusable with these slides. Although this experiment was reported for a gonochoric coral (*L. scutaria*), our preliminary data (not shown) suggested that the same results for these treatments on sperm concentration and motility held true for hermaphroditic coral (*M. capitata*), as well.

#### Effect of sample dilution on sperm motility

**Figure 3:**
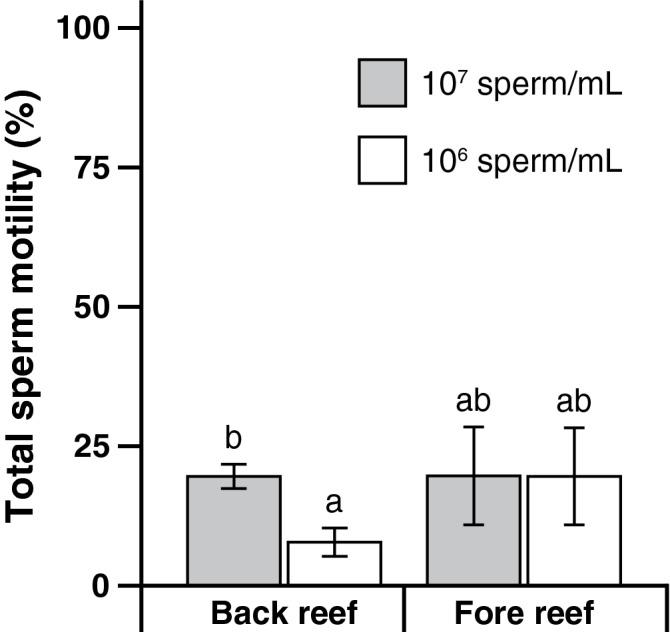
Total sperm motility was assessed with a phase microscope in samples from *Acropora hyacinthus* colonies collected from back and fore reef areas in Moorea, French Polynesia, diluted to concentrations of 10^6^ and 10^7^ cells/mL. Data are presented as mean ± SE and columns with the same letter are not significantly different (*P* < 0.05).

Frequently, we observed that coral sperm either reduced or stopped swimming after dilution with FSW. To quantitatively determine the effect of diluting sperm on the sperm motility analyses, we visually assessed sperm motility from samples at a concentration of 1 × 10^7^ cells/mL under the phase microscope, then re-evaluated the motility of the same samples after diluting them to a concentration of 1 × 10^6^ cells/mL. Gametes were collected from *A. hyacinthus* taken either from the back or fore reef in Moorea. Diluting sperm reduced sperm motility (mixed-model ANOVA, F_(1,14)_ = 5.19, *P* = 0.0388). Specifically, sperm motility for samples at 10^7^ cells/mL had a motility of 19.64% ± 2.93 (mean ± SE). In contrast, sperm diluted to 10^6^ cells/mL had a reduced motility of 11.52% ± 3.35 (Fig. 3; Supplementary Tables S6, S7). We noticed that the effect of sperm dilution might be different between the inner and outer reef colonies; we tested this hypothesis and found a significant interaction between reef sites (back vs. fore reef sites) and the dilution (mixed-model ANOVA, F_(1,14)_ = 5.08, *P* = 0.0408). Specifically, dilution reduced sperm motility on colonies from the back reef (19.62% ± 2.18 (mean ± SE) at 10^7^ cells/mL vs. 7.85 ± 2.54 cells/mL at 10^6^ cells/mL), but less so from those from the fore reef (19.67% ± 8.77 at 10^7^ cells/mL vs. 19.60 ± 8.71 cells/mL at 10^6^ cells/mL).

### Considerations for assessing sperm concentration

#### Effect of BSA on sperm concentration across three species

**Figure 4:**
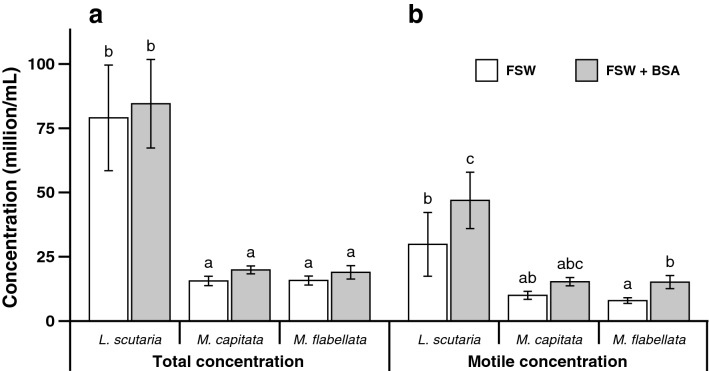
Computer-assisted sperm analysis (CASA) assessment of total (**a**) and motile (**b**) sperm concentrations in samples from *Lobactis scutaria* (n = 20), *Montipora capitata* (n = 9), and *Montipora flabellata* (n = 15) in the presence of filtered seawater (FSW) and filtered seawater with bovine serum albumen (FSW + BSA). Data are presented as mean ± SE, and in each panel (**a** and **b**), columns with the same letter are not significantly different (*P* < 0.05).

As with the flow cytometry trials with *L. scutaria* above, the addition of BSA trialled across three species did not affect sperm concentration overall. A small effect of BSA on total sperm concentration was detected by the ANOVA (Fig. 4a, mixed-effects ANOVA, F_(1, 43)_ = 11.35, *P* = 0.0016) but was not confirmed in the more conservative post hoc analyses (LS means post hoc pairwise comparisons). The total concentrations were different among the three examined species (mixed-model ANOVA, F_(2,41)_ = 22.91, *P* < 0.0001), with means of 81.80 ± 13.25 × 10^6^ cells/mL (mean ± SE), 17.73 ± 1.28 × 10^6^ cells/mL, and 17.34 ± 1.56 × 10^6^ cells/mL for *L. scutaria*, *M. capitata*, and *M. flabellata*, respectively (Supplementary Tables S4, S5).

Adding BSA did, however, increase the motile concentration. Similar to total motility and progressive motility (as shown in results above), BSA improved the average motile concentration from a range of 8‒30 × 10^6^ cells/mL to a range of 15‒47 × 10^6^ cells/mL for all three species (Fig. 4b, mixed-model ANOVA, F_(1, 43)_ = 52.82, *P* < 0.0001).

#### Comparison of instruments

**Figure 5:**
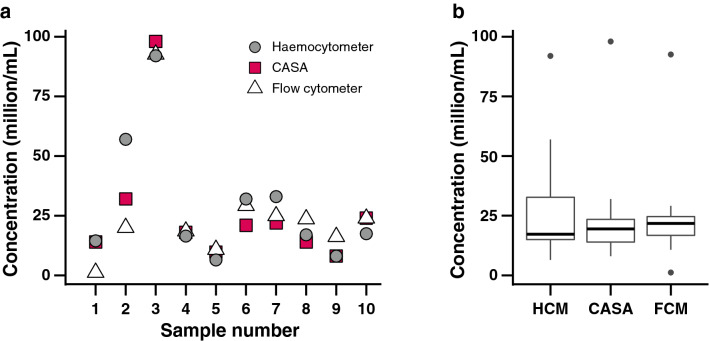
Sperm concentration in *Lobactis scutaria* samples (n = 10) measured using haemocytometer (HCM), computer-assisted sperm analysis (CASA), and flow cytometer (FCM). Data are presented individually (**a**) and as a box and whisker plot (**b**) showing median, sample range, and outliers (dots) for each assessment method.

We tested whether the measurement of sperm concentration by CASA matched conventional readings made with a haemocytometer and with a flow cytometer. Constrained by the limits of the analysis software, the sample concentration for CASA ranged from 5 × 10^7^ to 8 × 10^6^ cells/mL, whereas haemocytometer and flow cytometry ranged from 5 × 10^6^ to 1 × 10^6^ cells/mL. The values for all sperm concentration counting methods were plotted for each individual and a range for each individual and an average range across the counting methods was determined for each species. Sperm concentration was comparable among the three methods used (haemocytometer, CASA, and flow cytometer) to assess the total cell concentration (mixed-model ANOVA, F_(2, 18)_ = 0.64, *P* = 0.54; Fig. 5; Supplementary Tables S8, S9).

### Fertilisation and motile concentration

**Figure 6:**
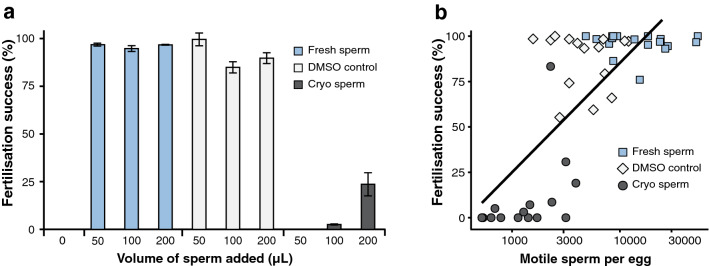
*Lobactis scutaria* reproduction. (**a**) When fresh sperm and fresh sperm + 10% DMSO were added to fresh eggs, the reproductive success was > 75% across all fresh sperm treatments. However, during cryopreservation the motile concentration was reduced by a factor of 10, thereby reducing the egg:sperm ratio to 1:5400 and 1:13,000 of motile sperm for the successful cryo trials. (**b**) Understanding the motile concentration of cryopreserved sperm and obtaining accurate egg:sperm ratios are critical for predicting robust fertilisation success with thawed sperm samples. This figure shows correlation between motile sperm concentration per egg (x-axis, logarithmic scale) and fertilisation success (y-axis) in eggs fertilised by fresh sperm in FSW, fresh sperm in 10% DMSO, and cryopreserved sperm in 10% DMSO. The correlation coefficient r between the two variables is 0.75 (*P* = 8.6 × 10^−10^). Fresh sperm samples from 6 males were pooled and mixed with individual females (n = 6). One and two data points are missing in the fresh sperm + 10% DMSO, 100 and 200 µL categories, respectively. Bars in panel **a** are SE.

Cryopreservation procedures are not always ideal and their results can change from spawning night to spawning night^14^; the post-thaw measurements of motility and percent motile sperm can be an order of magnitude lower than in the fresh sample. This can be compensated for by adding more of the cryopreserved sperm to the eggs to increase motile concentration per egg, but increasing cryoprotectant concentrations could negatively impact fertilisation due to toxicity. We examined how this increase in sperm volume might impact coral fertilisation success. Coral eggs were exposed to varying concentrations of fresh sperm, fresh sperm in DMSO, and frozen–thawed sperm in DMSO. Fresh sperm in FSW and in DMSO overall yielded higher fertilisation success than cryopreserved sperm (Fig. 6a); this was likely in part due to a higher concentration of motile sperm per egg used in the fresh sperm treatments, in comparison with the cryopreserved treatment (Table 1). Fertilisation success and motile sperm concentration per egg were strongly correlated (Pearson's correlation, r = 0.7498, *P* = 8.6 × 10^–10^), and are a key factor to consider when using cryopreserved sperm to produce successful offspring (Fig. 6b). Increasing the motile concentration of fresh sperm and fresh sperm + DMSO did not affect the mean fertilisation success. Thus, in these experiments cryoprotectant toxicity did not appear to negatively affect fertilisation success.

**Table 1:** Sperm motility and egg:sperm ratios for fresh, DMSO-treated, and cryopreserved sperm in three volumes (50, 100, and 200 µL) used for fertilisation.

	Fresh sperm	DMSO sperm	Cryo sperm
Total motility (%)	54	65	6
Motile concentration	2.4 × 10^7^ cells/mL	1.1 × 10^7^ cells/mL	2.0 × 10^6^ cells/mL
**Egg: motile sperm ratio**			
50 µL sperm	7.4 × 10^3^ cells/egg	2.8 × 10^3^ cells/egg	8.4 × 10^2^ cells/egg
100 µL sperm	1.4 × 10^4^ cells/egg	5.5 × 10^3^ cells/egg	1.2 × 10^3^ cells/egg
200 µL sperm	3.4 × 10^4^ cells/egg	9.6 × 10^3^ cells/egg	3.0 × 10^3^ cells/egg

## Discussion

The present study sought to identify the key factors affecting the accuracy and reproducibility of sperm quality analysis using CASA, and to establish analysis parameters that can be applied reliably across a range of coral species. Analysis of motility and concentration is usually the primary means of estimating the fertilising ability of a sperm sample. Traditionally, sperm motility is assessed by viewing the sample under a phase contrast microscope and estimating the proportion of sperm that are actively swimming; however, this subjective analysis can result in variability among operators, making comparisons between studies difficult. The use of CASA to provide rapid and objective assessment of sperm quality is well established in mammalian species^25,26^ and is increasingly used in aquatic species^27^. The use of CASA has been extended to certain invertebrate species with restricted spawning periods, as well as some for which induced spawning methods are available, meaning that there is a reliable supply of gametes for experimentation and refinement of analysis parameters. Unfortunately, none of these existing CASA protocols and settings is directly transferrable to coral sperm due to differences in motility characteristics, and the limited reproductive period of coral means that experiments are restricted to a brief annual window when spawning occurs. Moreover, coral sperm motility can vary greatly from night to night, even within the same colony. While the results in the present study were primarily collected during the 2019 and 2020 Hawaiian summers, the CASA analysis parameters used were the culmination of work conducted during annual spawning events over a 5-year period.

Initially, CASA did a very poor job of accurately measuring total and progressive motility because the sperm would stick to the CASA cassette, which has a proprietary coating. This was solved by adding BSA, caffeine, or BSA + caffeine to the sperm and FSW.

The problem of changing motility was not a problem with the CASA or the cassette, but rather a biological problem. The effect of dilution on coral sperm motility appeared to be greater in colonies that were exposed to a high thermal variance and irradiance, and that may have experienced thermal stress prior to the spawning season, either as an individual bleaching event or as an accumulation of stress over a longer period. Our data for the acroporid corals in Moorea demonstrated that a ten-fold dilution significantly reduced motility in sperm from back reef corals that had previously experienced elevated water temperatures and bleaching. Additionally, the size and age of the *A. hyacinthus* colonies should be considered. Back reef corals had much larger and older colonies than the fore reef colonies. The 2010 infestation of crown-of-thorns starfish, coupled with cyclone Oli in the same year, reduced coral cover on the outer reef to ~ 3%^28^. *L. scutaria* in Hawaii, which experienced bleaching and elevated water temperatures in 2014 and 2015, demonstrated a significant reduction in motility, sometimes to almost zero, in response to a tenfold dilution (Hagedorn et al., unpublished data). However, dilution did not appear to affect sperm from acroporid colonies from the fore reef location in Moorea. Preliminary observations of activated motility in acroporid colonies from the central region of the Great Barrier Reef in Australia in 2019 indicate that dilution impacted motile velocity (i.e., how fast the sperm were swimming) but did not reduce the overall motility (J.K. O’Brien and R. Hobbs, pers. comm.). Moreover, the decline in *L. scutaria* sperm motility observed in response to dilution was a relatively recent phenomenon, as sperm dilution for microscope assessment of motility was performed routinely in both *M. capitata* and *L. scutaria* in Hawaii without observable effects on motility prior to bleaching events in 2014 and 2015. Thus, the use of CASA could therefore provide an effective and novel means of monitoring the effects of ocean warming on reproduction in corals, and further research is required to investigate the relationship between thermal stress and sperm motility to understand the mechanisms of these changes.

A study by Morita et al.^29^ suggests one possible mechanism behind the dilution effect. These authors showed that sperm motility was regulated by proximity to eggs in three *Acropora* species, and that this response was species-specific. Sperm motility was increased by the proximity of conspecific eggs pre-fertilisation and decreased by their proximity post-fertilisation, presumably with the effects of reducing hybridisation and polyspermy, respectively. The dilution of the unknown regulatory substance(s) that are associated with the presence of eggs pre-fertilisation may account for our observed decreases in motility with increased sperm dilution. If correct, this would suggest that the duration of exposure to eggs in hermaphroditic species should be standardised in future studies that measure sperm motility. However, a dilution-associated reduction in motility is observed in gonochoric as well as hermaphroditic corals, which alternatively suggests that a sperm-associated (rather than egg-associated) factor may be responsible in whole or in part. In this case, antioxidants analogous to those present in mammalian seminal plasma, which affect the activity of reactive oxygen species^30^, may decline in effect with increasing dilution. The pathways producing such antioxidants could be impeded by bleaching stress, which would help to explain the observed dilution effect post-bleaching.

CASA was found to be a rapid and effective way to determine sperm concentration, and the values obtained were not significantly different from concentration measurements made using flow cytometry or haemocytometer counts. While haemocytometer count is a relatively simple and accurate means of determining cell concentration in a sample, it can be time-consuming as samples need to be prepared, loaded into the counting chamber, and allowed to settle before a reliable count can be made.

Although it is relatively simple to control sperm concentration in bundle-spawning hermaphroditic species, obtaining a suitable sample for CASA analysis from gonochoric species can be challenging. Sperm samples from these species are typically collected from the water column, so collecting a sample within the concentration range required for CASA without requiring subsequent adjustment depends very much on the skill of the person collecting. In the present study, sperm samples collected from *L. scutaria* were particularly susceptible to the effects of dilution after collection, so extreme care was required to ensure accurate comparisons between individuals. We therefore recommend that samples collected from gonochoric species should be performed by persons trained in sperm collection from that species and with a good understanding of sample requirements for CASA analysis to ensure accurate assessment and comparison of sperm quality parameters among individuals and populations.

CASA quickly measures one of the most important sperm assessment parameters—motile concentration. For coral, percent motile sperm is often reported, especially after cryopreservation^14^. However, for percent motility data to be useful it is important that it be placed in the context of sperm concentration, since a relatively low percent motility can still result in good fertilisation success if the concentration of motile cells is high enough, and a high percentage motility is meaningless if the concentration of cells is too low to achieve fertilisation. Motile concentration is particularly important for the effective use of cryopreserved sperm since a large proportion of cells is usually lost during the freezing and thawing process; even a sample with a relatively high percent motility may fail to fertilise if the actual number of motile sperm is below the threshold sperm-to-egg ratio required for fertilisation^11^. This is usually less of an issue with fresh sperm; however, it will likely become more important as the impact of ocean warming on reproduction in coral increases over the coming decades. The negative impact of coral bleaching and thermal stress on coral reproduction is well known^8,31^, and declines in sperm quality in response to prolonged thermal stress and bleaching have been documented in Hawaii^32^. Consideration of motile concentration and control of sperm-to-egg ratio may therefore become a crucial part of ex situ coral reproduction efforts for restoration^11^. We strongly recommend that the reporting of motile concentration be included in all future sperm CASA assessments for coral.

In our fertilisation trials, egg-to-sperm ratio was generally an order of magnitude higher in the fresh treatments than in the frozen–thawed treatments, compared to the corresponding volume of cryo sperm. However, the 200 µL frozen–thawed treatment had roughly the same egg-to-sperm ratio as the 50 µL DMSO sperm treatment, and only a quarter of the fertilisation rate. It is therefore likely that there is some additional degradation occurring in the frozen–thawed sample beyond what is captured by motility measurements. Nonetheless, the correlation found between motile sperm per egg and fertilisation success suggests that standardising the motile concentration of frozen–thawed sperm is likely to increase the reliability of fertilisation outcomes.

Our results indicate that the addition of BSA had a beneficial effect on the reliability of coral sperm motility assessment using CASA. A major issue early in the development of CASA analysis for coral sperm was the observation that in some samples, a large proportion of the sperm appeared to be vibrating in place rather than swimming forward. This was in contrast to what was observed when the same samples were viewed under phase contrast microscope using a regular glass slide and coverslip, and so was thought to be caused by some property of the well slides used for CASA analysis. This problem was largely overcome through the addition of BSA to the sample, which generally reduced the number of sperm that were vibrating in place while increasing the number that were moving forward during analysis. The addition of a protein component is common in diluents used for cryopreservation and assessment of sperm from mammalian species, but is often omitted in sperm from aquatic species. Since the development of the CEROS II system for CASA has predominantly occurred using mammalian sperm and diluents which typically contain some protein fraction, it is likely that sperm sticking is not an issue commonly encountered in most species for which CASA is used. Despite the apparent beneficial effect of BSA addition for CASA analysis in fresh sperm samples, we would caution against the addition of BSA to FSW as a sperm cryopreservation diluent for coral, as in preliminary experiments it was found to be detrimental to post-thaw survival due possibly to solution effects during freezing. Moreover, in our assessments of cryopreserved coral sperm using CASA we did not observe the same issue with sperm sticking in post-thaw samples. Our preliminary experiments suggest that this is due to the presence of cryoprotectant (dimethyl sulfoxide) in the post-thaw sample, which apparently has a similar effect to BSA in overcoming the stickiness of the well slides used for CASA.

It was noted during the present study is that there is a tendency for sperm to migrate toward the edges of the analysis wells over time. Since analysis should be performed on videos captured toward the centre of the well (i.e., away from the edges), delayed analysis of a sample after loading can result in an under-estimation of concentration and affect the reliability of motility assessments. If repeated analysis of a sample is required (e.g., to observe changes to motility over time), it is therefore essential to prepare a new well for each analysis since repeated analysis of the same well will provide inaccurate data.

Biobanking of coral sperm often requires concentration and motility assessments, processing, and cryopreservation of samples from dozens of individual colonies in a single night, so delays in assessment of concentration can have a significant effect on workflow efficiency and can result in degradation of samples over time as they await processing. The CASA protocols developed in the present study therefore have the potential to greatly increase the efficiency of sperm processing and cryopreservation in corals, enabling more samples of a higher quality to be secured in biorepositories.

The use of CASA will be essential to the application of sperm cryopreservation as a tool to secure genetic diversity and contribute to reef restoration efforts. As ex situ reproduction efforts to restore impacted reefs and to help coral to adapt to a changing climate increase in scope, so too will the importance of cryopreservation: it has been identified as a viable intervention for coral management by the U.S. National Academies of Sciences, Engineering, and Medicine^33^ and by the Australian Reef Restoration and Adaptation Program^34^. Cryopreservation can help with many aspects of reef restoration, including the reintroduction of genetics, broodstock development, and assisted gene flow between isolated populations^12^. Many of the interventions proposed for enhancing coral resilience to ocean warming and bleaching will rely on the use of gametes from valuable parent colonies collected or bred from isolated or genetically important populations, many of which may have diminished gamete quality. Technologies such as CASA and sperm cryopreservation can play a crucial role in ensuring that these valuable samples are used effectively and efficiently in restoration programs and that they help to minimise the loss of genetic diversity on the world’s coral reefs.

## Methods

### Coral collection and husbandry

*Hawaii*—Three species of coral were collected in Hawaii: whole individuals of the solitary coral *Lobactis scutaria* (n = 30 in 2019 and n = 14 in 2020) and fragments, approximately 10 cm in length, of the colonial *Montipora capitata* and *M. flabellata* (n = 9 and n = 15, respectively, in 2019). Collections were made from the shallow patch reefs in Kaneohe Bay, Hawaii in May and June of 2019 and in June of 2020, taking from several distinct reefs to yield as much genetic diversity as possible. All corals were held in open seawater systems drawing directly from Kaneohe Bay with natural temperature and light exposure throughout the spawning season. All collection was carried out under permits from Hawaii’s Department of Land and Natural Resources (Special Activity Permits 2019–2016 and 2021–2033). Although no IACUC or other institutional ethical approval is required for invertebrate care, their husbandry was maintained at the highest standard. Water conditions and colony health were monitored daily, and corals were returned to the reef following reproduction**.**

*French Polynesia*—Colonies of *Acropora hyacinthus* were collected prior to spawning in Moorea, French Polynesia in October 2018. Entire colonies (20–30 cm in longest dimension) were sampled from several back and fore reef locations, approximately three to five days before the spawning around the spring full moon. Back reef is defined as inside the lagoon near to the crest, but far from the shore. Fore reef is defined as outside the lagoon from the reef crest onwards. *A. hyacinthus* is only present in back and fore reefs. These collected corals were maintained in running seawater at the Centre de Recherches Insulaires et Observatoire de l’Environnement (CRIOBE) in Moorea, French Polynesia. A numbered scientific research permit was obtained for the entire station each year, but no collection permit is currently required to collect coral in French Polynesia. Corals were placed back at their respective reefs following spawning.

### Gamete collection

Methods for gamete collection differed between species to account for reproductive strategy: hermaphroditic and gonochoric corals require different techniques^14,35^. Most hermaphroditic corals release positively buoyant bundles of tightly packed eggs and sperm that can be collected from the water surface. In gonochoric corals, females release slightly negatively buoyant eggs, and males release sperm that is rapidly diluted in the surrounding water. For such corals, sperm must be sampled quickly and directly from the corner of the polyp’s mouth to capture an adequate sperm concentration.

*Lobactis scutaria*, a solitary, gonochoric coral, spawns from June to September in the early evening two to four days after the full moon^36^. During the afternoon immediately prior to spawning, individuals were isolated in separate bowls; spawning started between 4:30 and 6:30 p.m. Female corals that released eggs were set aside and not used in this study. Sperm, however, was collected immediately upon release from the mouth. Plastic transfer pipettes were used to remove sperm into 15-mL or 50-mL tubes at the highest possible concentration (2019), or at a target concentration specified below (2020). Sperm samples were transported immediately to the laboratory for motility and concentration assessment.

*Acropora hyacinthus*, *Montipora capitata*, and *M. flabellata* are simultaneous hermaphroditic bundle-spawners, as is typical of acroporids^37^. *A. hyacinthus* in Moorea spawns following the full moon in October and November, between 10:00 and 10:45 p.m.^38^. In Hawaii, *M. capitata* spawns in June and July, in the late evening on the new moon and up to two nights thereafter^39^, and *M. flabellata* spawns in parallel with *M. capitata*. Approximately an hour before each acroporid’s spawning event, colonies were isolated from each other in plastic containers to prevent mixing of egg–sperm bundles.

*A. hyacinthus* started setting at 7:30 p.m. and spawning began at 10 p.m. The egg-sperm bundles were collected from the water surface with transfer pipettes into 50-mL centrifuge tubes at a concentration of 5 mL of bundles in 5 mL of 0.22-µm-filtered seawater (FSW) for a total volume of 10 mL at approximately 1 × 10^9^ cells/mL. Bundles were allowed to break up over approximately one hour. *M. capitata* and *M. flabellata* both began setting and spawning around 9:00 p.m. and their egg-sperm bundles were collected from the water surface using either 200-µL pipettes with cut-down tips (to prevent damage to the bundles) or with transfer pipettes. Bundles were placed into 50-mL centrifuge tubes with 50 bundles over 10 mL of seawater, producing a sperm concentration of approximately 10^7^ cells/mL once the bundles broke apart. *M. capitata* has a toxin in its eggs, released by disturbance or damage to the eggs, which can negatively affect sperm motility^35^. Bundles were therefore allowed to break apart in tubes without agitation over approximately 30 min. Once the bundles were completely broken apart, concentrated sperm was removed from the bottom of the tube with a pipette for assessment.

### Microscope setup and CASA settings

Sperm assessment was conducted with a CEROS II CASA system (Hamilton Thorne, Boston, MA). The CASA was connected to either of the two following microscopes: the Olympus BX41 (10 × objective [Olympus UPlan FL N, 10 × /0.30 Ph1, infinity/0.30/FN 26.5], 1 × c-mount and no light source filter) or the Olympus BH-2 (10 × objective [Olympus Japan 133,781, 0.30 160/0.17], 0.7 × c-mount, and green light source filter). Our system differed from the standard Hamilton Thorne CASA platform in that we used our own microscopes in modified darkfield setups, rather than the phase microscopes often supplied with CASA systems (the typical CASA setup uses a 10 × phase 1 objective with a condenser correspondingly set to phase 1). Our condenser and software both had to be adjusted carefully for consistent detection by the CASA software.

CASA always needs to be adjusted for a new species to allow cell detection and frame-to-frame tracking. Head size, brightness, sperm velocity, and blur (owing to depth of field) had to be accounted for. See the Results and Supplementary Table S8 for the specific settings that we used for coral sperm.

### CASA cassette loading and video captures

Standard Count 4 Chamber Slides (3 µL volume, 20 µm depth, REF SC20-01-04-B, Leja, Netherlands) were used for measuring sperm parameters with the CASA. The slides were loaded with 4 µL of sperm and immediately once the sample had wicked through the length of the viewing chamber, excess fluid was completely removed with a Kimwipe. In the CASA software, distinct video fields were captured for each sperm sample until at least five fields were captured and at least 200 sperm cells were measured. The videos were consistently visually checked by the operator as they were being captured, to detect any unusual readings such as false motility produced if the microscope platform was inadvertently bumped, or uniform flow in the chamber due to improper excess fluid removal before video capture.

### Use of BSA for sperm motility assessments with CASA

From the beginning of sperm cryopreservation studies, many species’ cryopreservation protocols included some kind of protein in their sperm diluent^40^ to prevent the sperm from adhering. Initially the coral protocols only used FSW with the sperm; however, during our sperm assessments, we noticed that the sperm were intermittently sticking to the CASA cassettes. We introduced BSA into our sperm analysis protocol to test whether it could prevent sperm from sticking on slides, and whether it would improve consistency and sperm motility^41^. To verify whether adding BSA improved sperm motility in coral sperm, we used two treatments (FSW and FSW + BSA) and examined sperm samples from various coral species and colonies [*L. scutaria* (n = 20)*, M. capitata* (n = 9) and *M. flabellata* (n = 15)].

Bundles from the *Montipora* species were collected in 15-mL tubes to target a sperm concentration of approximately 1‒3 × 10^7^ cells/mL. This could be assessed on the CASA without too much further dilution of the sperm (see gamete collection for the correct number of bundles per tube to reach this cell concentration target). To create the FSW + BSA treatment, a 0.1% dilution of this initial sperm concentration (already suspended in FSW) was made with BSA [10 µL of a BSA 30% stock (Sigma-Aldrich product # A9576-50ML, 30% solution in DPBS, Batch number SLBT8229) with 990 µL FSW]. The sperm–BSA solution was mixed gently. Both the FSW and FSW + BSA treatments were analysed with CASA within 2 min.

To examine this process in another species, *L. scutaria* sperm was treated similarly, except it was collected from the mouth of the coral as it was expelled. The difference with the above species is that the FSW treatment and the FSW + BSA treatments were both diluted 0.1% either with the BSA solution or FSW before being assessed by CASA.

### BSA and caffeine tests

Sperm from each *L. scutaria* individual were collected at a target concentration of approximately 1‒3 × 10^7^ cells/mL and were exposed to four treatments to determine the effects on sperm motility and concentration. These were: (1) FSW, (2) 0.3% Bovine Serum Albumin (30% BSA, Sigma Aldrich, St. Louis, MO), (3) 12 mM caffeine (Sigma Aldrich, St. Louis, MO), and (4) 0.3% BSA + 12 mM caffeine. Caffeine addition was based on a protocol developed in sperm from *Acropora* corals (Dr Justine O'Brien, Taronga Conservation Society, Australia, personal communication). For each sample, sperm were diluted at a ratio of 9:1 (sperm : activation solution) in a single step. Sperm motility and concentration were assessed with CASA and a flow cytometer. For each of the four treatments, sperm was aliquoted into an Eppendorf tube with 90 µL sperm and 10 µL treatment solution, mixed gently, and tested after 5 min. For each sample treatment, 4 µL was loaded into a Leja Standard Count 4-Chambered Slide (SC 20–01-04–8, Leja Products, The Netherlands) and immediately assessed. The CASA’s built-in calculation for dilution was used.

After the initial motility and concentration were obtained from the FSW (control) CASA measurements, each individual’s original, untreated sperm sample concentration was also assessed with flow cytometry (BD Accuri C6 Plus, BD Biosciences, San Jose, CA). Using the CASA sperm concentration from the FSW treatment, the original sperm sample was diluted with FSW to a target of 5 × 10^6^ to 1 × 10^7^ in 500 µL. These samples were diluted in half with FSW, analysed on the flow cytometer by collecting 10 µL at a rate of about 5,000 events/sec, and gated for forward- and side-scatter to exclude debris.

### Effect of sample dilution on sperm motility

In comparing how the CASA motility assessments aligned with our years of qualitative coral sperm assessment^14^, we noted inconsistencies in our use of the CASA. For example, total motility percentages differed substantially from what we perceived by eye, and not in any consistent manner. We struggled with these inconsistencies and decided to test whether diluting our sperm samples had an effect on the motility, given that assessments done with CASA and assessments done by eye with a phase microscope often required different optimal concentration ranges. The working range for the CASA to read and analyse sperm is approximately 8 × 10^6^ to 5 × 10^7^ cells/mL, and ideally 1 × 10^7^ to 3 × 10^7^ cells/mL. This is usually too high to read accurately by eye with a phase microscope, which is better suited to a range of 1 × 10^6^ to 1 × 10^7^ cells/mL.

To investigate changes in sperm motility when diluting our samples, we used sperm from *A. hyacinthus* in French Polynesia, collected at a concentration of 1 × 10^9^ cells/mL. The samples were then diluted to 1 × 10^7^ and 1 × 10^6^ cells/mL for comparative motility assessments under a phase microscope. We investigated sperm from 16 colonies: 11 came from the back reef, exposed to higher thermal variance and irradiance due to the shallow depth (1–1.5 m), and five came from the fore reef (10 m in depth), less subject to thermal stress and governed by much greater mixing and thermal stability from oceanic waters^42^.

### Comparison of instruments

The traditional method to determine sperm concentration is to count the number of cells in a fixed volume on a haemocytometer^43^, but CASA enables rapid sperm counting, reducing the need for haemocytometer counts. Because we had issues with verifying sperm motility between CASA and standard microscope measures, we wanted to ensure that concentrations determined with CASA were comparable to those determined with either a haemocytometer or flow cytometer.

We validated the sperm concentration measurements of the CASA and the flow cytometer against a haemocytometer using sperm from the mushroom coral *L. scutaria* in Hawaii (n = 10). Sperm samples for analyses were taken from the same sperm expulsion (10-mL aliquot of sperm from each *L. scutaria* individual) and put on ice for counting. The sperm concentration was diluted to ~ 10^7^ cells/mL and measured by CASA shortly after spawning, and the flow cytometry assessments were conducted shortly thereafter. For the flow cytometer, a 50-µL aliquot was removed from the same CASA-assessed sperm sample and diluted at a ratio of 1:9 by adding 450 µL of FSW, creating a sample at ~ 10^6^ cells/mL. The diluted sample was mixed well to ensure even suspension of cells and the sample was immediately analysed on a BD Accuri C6 flow cytometer by collecting 10 µL at a flow rate of 14 µL/min. The total event count was viewed on a forward scatter vs. side scatter plot using CFlow Plus software (Version 1.0.23.1, BD Accuri) and the sperm population was gated to exclude debris. Sperm concentration was then calculated by multiplying the event count within the gate by the dilution factor, and then by 100 to provide a concentration per mL.

Haemocytometer counts were done 2‒3 h after CASA and flow cytometer analysis, using the 10^6^ cells/mL sample from the flow cytometer. The sample was mixed thoroughly and 10 µL was pipetted onto the haemocytometer. Counts were done at least twice and by two different observers. The concentration was calculated with the following equation:

$$ Concentration = CC \times N \times CD \times D\;cells/mL $$

with *CC* = cell count, *N* = number of haemocytometer cells (minimum 3, with each haemocytometer cell composed of a grouping of 16 smallest squares), *CD* = chamber depth, and *D* = dilution.

### Fertilisation and motile concentration

*Lobactis scutaria* eggs were collected (n = 6) and 28–90 eggs were placed into 5 mL FSW. Sperm samples (n = 6) were collected at a concentration of 2 to 4 × 10^7^ cells/mL and pooled. These were added to the eggs in four treatments: (1) no sperm added; (2) 50, 100 and 200 µL fresh sperm; (3) 50, 100 and 200 µL fresh sperm diluted 1:1 with 20% DMSO; and (4) 50, 100 and 200 µL of cryopreserved and thawed sperm. Cryopreservation was done according to the methods of Hagedorn et al.^14^. Fertilisation success was assessed 3–6 h after fertilisation by counting cleaved and uncleaved eggs in each sample.

### Statistical analyses

The effect of adding BSA to the sperm for motility and concentration assessments with CASA was investigated in three species (*L. scutaria*, *M. capitata*, *M. flabellata*) with a mixed-model ANOVA with species and addition of BSA as fixed factors, and sample ID as random factor. Concentration data were log-transformed to meet ANOVA assumptions. The effect of sperm dilution on sperm motility was investigated for *A. hyacinthus* with a mixed-model ANOVA with sperm concentration and reef site (fore reef or back reef) as fixed factors, and sample ID as random factor. To determine whether the sperm concentration was comparable with different methods (haemocytometer, CASA, and flow cytometer), a one-way ANOVA with sperm concentration as fixed factor and sample ID as random factor was performed on sperm from *L. scutaria*. Normality of residuals was verified visually and/or with a Shapiro‒Wilk normality test. Homoscedasticity and independence were verified visually by plotting the model residuals against the fitted model. Where relevant, post hoc tests were conducted with least square means pairwise comparisons with Tukey adjustment for multiple comparisons. To determine the relationship between fertilisation success and motile sperm concentration per egg, a linear regression was plotted and a Pearson's correlation test was conducted. A plot of fertilisation success and motile sperm concentration per egg revealed a logarithmic trend; therefore, motile sperm concentration per egg was log-transformed for the statistical tests and was plotted on a logarithmic scale for the figure. Normality assumptions were verified by running a Shapiro–Wilk test on the residuals of the linear regression. All statistical analyses were conducted using R^44^, with the R packages car^45^, rcompanion^46^, lsmeans^47^, multcomp^48^, multcompView^49^, nlme^50^, and ggplot2^51^.

## Supplementary Information

Supplementary Information.

## Data Availability

The datasets generated and analysed during the current study are available from the corresponding author on reasonable request.
