# Supplementary material for: Assessing coral sperm motility
Source: Sci Rep. 2021 Jan 8;11:61. doi: 10.1038/s41598-020-79732-x (PMC7794428; doi:10.1038/s41598-020-79732-x)
Supplement: Supplementary file 1 — Supplementary Information. [file 41598_2020_79732_MOESM1_ESM.pdf]

Supplementary Information to "Assessing coral sperm motility"

Nikolas Zuchowicz, Jonathan Daly, Jessica Bouwmeester, Claire Lager, E. Michael Henley, C. Isabel Nuñez Lendo, and Mary Hagedorn

I. Methods: Adjustments specific to our microscopes

The typical CASA setup uses a 10× phase 1 objective with a condenser correspondingly set to phase 1. Our system differed from the standard Hamilton Thorne platform in that we used our own microscopes (Olympus BX41 and BH-2 in modified darkfield setups), rather than the phase microscopes often supplied with CASA systems. Our condenser and software both needed to be adjusted carefully for consistent detection by the CASA software. In our setup, we found that a clear, computer-readable image with white sperm on a black background may be obtained with the condenser set to darkfield. This makes it possible to use CASA with a non-phase objective. In our case, we had phase settings available on our condenser, and we found that the phase 3 light cone was an improvement on the image produced by the darkfield light cone. This is effectively a clearer darkfield image than the usual darkfield setting. We believe that this is a matter of the phase 3 light cone being narrower and better collimated along its angled beam paths than the darkfield cone, while still being broad enough not to enter the 10× objective directly. In this setup, sperm tails are not clearly visible; however, this did not present any difficulty in detection by the CASA algorithm, with the proviso that "static tail detection" must be set to false – otherwise only moving cells will be counted in calculations of motility and concentration, which will lead to severe overestimates of motility and underestimates of concentration. The condenser was adjusted to the correct height by switching to brightfield, closing down the field diaphragm, manipulating the condenser height to bring the iris leaves into focus, opening the

field diaphragm back up to just larger than the CASA camera field of view, and switching back to phase 3.

The BX41 is in general a dimmer microscope than the BH-2. For this reason, a green field aperture filter, which is helpful in visually sharpening the edges of sperm heads but which reduces the intensity of light transmitted to the camera, was employed on the BH-2 but not on the BX41. The BH-2 has older, nonplanar objectives that led to obvious blurriness and distortion of individual sperm heads around the edges of the image. Application of the green filter helped to mitigate this, but it was also necessary to reduce the minimum acceptable head elongation ("Elongation Min") from 50% (as on the BX41) to 30% to permit consistent detection of all sperm heads.

Supplementary Table S1: Hamilton Thorne CEROS II CASA setup for use with coral sperm on the BX41 and BH-2 microscopes with 10× objectives in darkfield configuration.

Parameter	BX41 settings	BH-2 settings	Notes
Analysis Limits			
Min Motility	0	0	
Min Progressive Percent	0	0	
Min Total Count	200	200	Our minimum capture standard was (200 sperm AND 5 fields).
Calibration			
Objective Mag X	1.2	0.83	
Objective Mag Y	1.2	0.83	
Camera			
Exposure (ms)	16	2	The BX41 is in general a darker microscope, requiring longer exposure and greater digital gain.
Gain	300	1	
Integrate Enabled	False	False	
Integrate Time (ms)	500	500	
Cell Detection			
Elongation Max (%)	100	100	
Elongation Min (%)	50	30	The older BH-2 shows some blur and elongation of sperm heads at the edge of the view field, hence broader acceptable elongation settings.
Enable Advanced Tail Detection	False	False	
Head Brightness Min	50	60	
Head Size Max (μm^2)	150	150	
Head Size Min (μm^2)	5	5	
Static Tail Filter	False	False	If set to true, only motile sperm will be detected, since our setup did not allow visualization of tails.
Tail Brightness Min	255	255	This prevents the software from detecting any pixels as tails.
Tail Min Brightness Auto Offset	8	8	
Tail Min Brightness Mode	Manual	Manual	
Chamber			

Capillary Correction	1.26	1.26	This correction factor corresponds to the slide load time (2.6 sec) for coral sperm in seawater.
Chamber Depth (µm)	20	20	
Chamber Type	Capillary	Capillary	
Illumination			
Histogram Smooth Width	0	As BX41	Photometer min/max was not used in our setup.
Max Photometer	30		
Min Photometer	20		
Kinematics			
Cell Travel Max (µm)	10	As BX41	
Enable Motile Static Collision Avoidance	False		
Motile cells require a tail	False		
Motile Require Tails Max VSL (µm/s)	0		
Progressive STR (%)	0		
Progressive VAP (µm/s)	80		This corresponds to a qualitative (visual) threshold for highly motile sperm; some species have a maximum VAP exceeding 200 µm/s.
Slow VAP (µm/s)	20		This corresponds to a qualitative (visual) threshold between sperm idly circling or twitching vigorously (slow) and sperm with meaningful forward movement (motile).
Slow VSL (µm/s)	0		
Static Algorithm	Width_Multiplier		This permits twitching sperm to be counted as slow motile rather than static.
Static VAP (µm/s)	0		
Static VSL (µm/s)	0		
Static Width Multiplier	0.8		Twitching sperm whose movement exceeds 80% of their head diameter are counted as slow motile.
Morph			
DMR Confidence (%)	1	As BX41	No morph data were studied.
DMR Droplet to tail end Max (µm)	1		
DMR Tail Length Max (µm)	0		

Droplet Confidence (%)	1		
Droplet Distal Distance Min (µm)	0		
Droplet Proximal Head Length (µm)	0		
Min Tail Length (µm)	0		
Morph Normal Minimum Percentage	0		
Tail Bend Angle Averaging Length (µm)	0		
Tail Bending Angle Rate Min (°/µm)	1		
Tail Bent Confidence (%)	1		
Tail Coiled Angle Min (°)	1		
Tail Coiled Confidence (%)	1		
Tail Confidence (%)	1		
Viadent Category			
Viadent Fluorescing Sperm	NonViable	As BX41	
Video Capture			
Frame Capture Speed (Hz)	60	As BX41	
Frame Count	45		

II. Effect of BSA on sperm characteristics

Supplementary Table S2: Summary of effect of added BSA on sperm motility in *L. scutaria*, *M. capitata*, *M. flabellata*

	**Total Motility (%)**		**Progressive Motility (%)**	
	FSW	FSW + BSA	FSW	FSW + BSA
L. scutaria				
N	20	20	20	20
Mean	37.3	57.1	15.7	35.7
Std Error	4.2	4.4	3.4	5.1
M. capitata				
N	9	9	9	9
Mean	61.7	76.5	57.8	73.4
Std Error	4.0	4.9	4.2	5.4
M. flabellata				
N	15	15	15	15
Mean	50.5	75.5	43.3	72.6
Std Error	5.1	4.7	6.1	4.8

Supplementary Table S3: Summary of the mixed-model ANOVA on the effect of added BSA on sperm motility, total and progressive

	**Percent Total Motility**[#]			
	DFn	DFd	F-value	p-value
Species	2	41	7.5905	0.0016
BSA	1	43	57.6131	<0.0001

	**Percent Progressive Motility**[#]			
	DFn	DFd	F-value	p-value
Species	2	41	26.1471	<0.0001
BSA	1	43	49.4906	<0.0001

[#]Based on Type II sum of squares

Supplementary Table S4: Summary of effects of added BSA on sperm concentration in *L. scutaria*, *M. capitata*, *M. flabellata*

	Total Concentration (million cells/ml)		Motile Concentration (million cells/ml)	
	FSW	FSW + BSA	FSW	FSW + BSA
L. scutaria				
N	20	20	20	20
Mean	79.1	84.5	29.8	46.9
Std Er.	20.6	17.2	12.4	11.0
M. capitata				
N	9	9	9	9
Mean	15.6	19.9	10.0	15.2
Std Er.	1.9	1.5	1.6	1.6
M. flabellata				
N	15	15	15	15
Mean	15.7	18.9	7.9	15.1
Std Er.	1.7	2.6	1.1	2.5

Supplementary Table S5: Summary of the mixed-model ANOVA on the effect of added BSA on total sperm concentration and motile sperm concentration

	Total Sperm Concentration[#]			
	DFn	DFd	F-value	p-value
Species	2	41	22.9124	<0.0001
BSA	1	43	11.3464	0.0016

	Motile Sperm Concentration[#]			
	DFn	DFd	F-value	p-value
Species	2	41	8.8466	0.0006
BSA	1	43	52.8221	<0.0001

[#]ANOVA conducted on log-transformed data; based on Type II sum of squares

III. Effect of Sperm Dilutions on Sperm Motility

Supplementary Table S6: Summary of the effects of sperm concentration/dilution and reef site on sperm motility observed under the microscope, in *Acropora hyacinthus*

Reef: ***Concentration:***	**Back Reef** **10^7**	**Back Reef** **10^6**	**Fore Reef** **10^7**	**Fore Reef** **10^6**
A. hyacinthus				
N	11	11	5	5
Mean	19.62	7.85	19.67	19.60
Std Error	2.18	2.54	8.77	8.71

Supplementary Table S7: Summary of the mixed-model ANOVA on the effects of sperm concentration/dilution and reef, on sperm motility, in *Acropora hyacinthus*

	**Percent Total Sperm Motility**			
	DFn	DFd	F-value	p-value
Site	1	14	0.920331	0.3537
Dilution	1	14	5.194837	0.0388
Site×Dilution	1	14	5.079666	0.0408

IV. Assessing Total Sperm Concentration with Haemocytometer, CASA, and Flow Cytometer

Supplementary Table S8: Summary of the effect of methodology (CASA, haemocytometer, or flow cytometer) on total sperm concentration in *L. scutaria*

	**HCM**	**CASA**	**FCM**
N	10	10	10
Mean	29.41	26.09	26.09
Std Error	8.41	8.30	7.81

Supplementary Table S9: Summary of the mixed-model ANOVA on the effect of methodology to assess total sperm concentration

	**Total Sperm Concentration[#]**			
	DFn	DFd	F-value	p-value
Methodology	2	18	0.64	0.540
